# Toward an experimental account of argumentation: the case of the slippery slope and the ad hominem arguments

**DOI:** 10.3389/fpsyg.2014.01420

**Published:** 2014-12-15

**Authors:** Marco Lillo-Unglaube, Andrés Canales-Johnson, Gorka Navarrete, Claudio Fuentes Bravo

**Affiliations:** ^1^Center of Argumentation and Reasoning Studies, Universidad Diego Portales, Santiago, Chile; ^2^Laboratory of Cognitive and Social Neuroscience, UDP-INECO Foundation Core on Neuroscience, Universidad Diego Portales, Santiago, Chile; ^3^Cognition and Brain Sciences Unit, Medical Research Council, Cambridge, UK

**Keywords:** argumentation theory, Bayesian models, similarity judgment, slippery slope argument, ad hominem argument

## Abstract

Argumentation is a crucial component of our lives. Although in the absence of rational debate our legal, political, and scientific systems would not be possible, there is still no integrated area of research on the psychology of argumentation. Furthermore, classical theories of argumentation are normative (i.e., the acceptability of an argument is determined by a set of norms or logical rules), which sometimes creates a dissociation between the theories and people’s behavior. We think the current challenge for psychology is to bring together the cognitive and normative accounts of argumentation. In this article, we exemplify this point by analyzing two cases of argumentative structures experimentally studied in the context of cognitive psychology. Specifically, we focus on the slippery slope argument and the *ad hominem* argument under the frameworks of Bayesian and pragma-dialectics approaches, respectively. We think employing more descriptive and experimental accounts of argumentation would help Psychology to bring closer the cognitive and normative accounts of argumentation with the final goal of establishing an integrated area of research on the psychology of argumentation.

## TOWARD AN EXPERIMENTAL ACCOUNT OF ARGUMENTATION

Argumentation is a crucial component of our lives since in the absence of rational debate our legal, political, educational, and even scientific systems would not be possible (Mercier and Sperber, 2011). Although psychology has studied several aspects of argumentation, such as its role in social engagement (Means and Voss, 1996), in learning and education (Asterhan and Schwarz, 2007, 2009; Mercer, 2009; Howe, 2010), and in the construction of knowledge (Mason and Santi, 1994; Leitão, 2000, 2008; Schwarz, 2009), there is still no integrated area of research on the “psychology of argumentation” (Hornikx and Hahn, 2012). Recently, Hornikx and Hahn (2012) have employed this concept for encapsulating both theoretical and experimental accounts that mutually inform separate research communities studying human reasoning and argumentation.

Furthermore, although classical theories of argumentation have been devoted to understanding argumentative processes both in academic and daily-life contexts, there is no common theoretical ground between these theories. For instance, rhetoric considers argumentation to be a tool for persuading the audience, whereas dialectics consider argumentation to be the quintessence of a critical discussion aiming to determine the acceptability of a particular stance or point of view (Wenzel, 1990). Despite these theoretical discrepancies, for both rhetoric and dialectics the acceptability of an argument is determined by a set of norms or logical rules which allow classifying an argument as veridical or fallacious, i.e., the so-called *normative* approaches for argumentation.

We think the current challenge in front of psychology is to bring together the cognitive and normative accounts of argumentation. In order to achieve this, we claim that the psychological mechanisms of argumentative processes should be investigated by employing more descriptive and experimental accounts. In line with this idea, recent work has started to examine empirically the descriptive, psychological aspect of classical argumentative fallacies. In particular, modern approaches for studying argumentation such as Bayesian theory (Hahn and Oaksford, 2007; Corner and Hahn, 2009; Corner et al., 2011), the pragma-dialectical account (van Eemeren and Grootendorst, 2004; van Eemeren et al., 2009, 2012), epistemic vigilance (Sperber et al., 2010), and evolutionary psychology (Sperber and Mercier, 2012), have proposed plausible explanations for the mechanisms and cognitive aspects of argumentation in more ecologically valid contextual accounts. In this article, we show how these descriptive approaches shed light onto the psychological mechanisms of argumentation.

Here we analyze experimental evidence of two classical argumentative structures. Specifically, we focus on the Bayesian analysis of the slippery slope argument and the pragma-dialectical analysis of the *ad hominem* argument. We think that further experimental research in the area is needed to increase the dialog between argumentation theory and cognitive psychology and thus provide a step toward an experimental account of argumentation.

## CASE 1: THE SLIPPERY SLOPE ARGUMENT

The slippery slope argument is an argument from consequences traditionally conceptualized as an informal fallacy (Walton, 1992). The argument starts by considering an execution of a seemingly harmless action. The argument exhibits how the implementation of the action would inevitably lead to an undesired or detrimental consequence. Then, a conclusion is reached that aims to avoid the undesired consequence. Here is an example of a slippery slope argument:

“The government should not negotiate with terrorists (1). Once the government starts considering terrorists as valid interlocutors (3), we will start having dozens of new terrorist attacks (2).”

We can see from this example that the structure of a slippery slope argument can be defined by three core aspects: (1) an initial decision intuitively acceptable; (2) a “case” or “situation” evaluated as unacceptable or dangerous; and (3) a process or mechanism by which violating the initial decision would facilitate the occurrence of that “case” or “situation” (Rizzo and Whitman, 2003).

In argumentation, the structure of the slippery slope argument has raised the question of its highly successful implementation in contexts in which a subject or a group of subjects attempts to persuade the audience in favor of an argument even when the argument or its usage are incorrect. In particular, cognitive psychology has initiated the investigation of the mechanisms underlying persuasiveness of the slippery slope argument by employing the cognitive concept of similarity and statistical tools from Bayesian theory (Corner et al., 2011).

### SIMILARITY AND THE SLIPPERY SLOPE ARGUMENT: A BAYESIAN APPROACH

Similarity is the cognitive process of perceiving objects as a global unity when they share similar physical characteristics and as different objects when they do not (Tversky, 1977). Thus, similarity represents one of the main “grouping” principles in psychology. The classical approach in cognitive psychology assumes that concepts can be represented in a common problem space in which they are depicted as points in that space. Then, similarity is operationally defined as the distance between concepts (i.e., points) in that space. Objects that are psychologically more similar would be closer than ones that are dissimilar (Tversky, 1977).

Recent experimental evidence from the study of informal fallacies and decision making have shed light on the psychological mechanisms of the slippery slope argument by employing the notion of similarity (Hahn and Oaksford, 2007; Corner and Hahn, 2009; Corner et al., 2011). Specifically, this line of research has tested the hypothesis that the more similar the antecedents in an argumentative chain are, the more persuasive (or slippery) the slope will be. In other words, the mechanism underlying the acceptance of a slippery slope argument would be related to the degree of similarity between the antecedents of the argumentative structure.

In the last years, this hypothesis has been tested under the Bayesian account of argumentation (Corner et al., 2011). This approach considers fallacies as inductive conditional arguments in which the strength of the argument depends on the probability of the precedent actually preceding the consequent. These probabilities are determined by previous experience. In the case of the example described above, the argument is convincing when the conditional probability of the government negotiating with terrorists (i.e., antecedent A) is high due to the increase in terrorist attacks (i.e., consequent C). Then the calculation of the probability is *P*(C|A). Thus, the conclusion consists of negating the antecedent since the antecedent has a negative utility. The underlying mechanism fixing the relevant probabilities for the model, i.e., *P*(C|A), follows the continuous change of boundaries—as in distance in similarity between the categories. Then, accepting the antecedent in a slippery slope argument makes us prone to accept the consequence. In other words, accepting one element (i.e., antecedent—talking to terrorists) as part of a category (i.e., the consequence—terrorist attacks) would lead us to accept another element (i.e., negotiating) as part of the same category.

Corner et al. (2011) proposed a psychological mechanism of the slippery slope argument consisting of the re-appraisal of category boundaries based on the similarity or closeness between items in conceptual space. The rationale is that classifying an item *a* under a category *F* increases the probability that a further item *b* will be classified under the same category *F*. The authors employed a type of argument that allows to calculate similarity in the context of a decision making task. Thus, the experiment comprised of deciding whether action A should be carried out or not. In one example, participants had to decide whether an area is eligible or not for the status of “Outstanding Natural Beauty” by considering its inhabiting species. For instance:

Scarathon is home to 224 species of large animals.

Sellenfeld is home to 179 species of large animals.

Decision: Eligible for Area of Outstanding Natural Beauty status.

In these experiments, participants were asked to make a categorization decision of their own (i.e., whether Sellenfeld was eligible for the Outstanding Natural Beauty status), based on the information they had just read. The experiments were designed to demonstrate that the evaluation of a slippery slope argument is directly related to the re-appraisal of categorical boundaries. Specifically, the information was presented either as a categorization task, or as decision-making task. Experimenters showed that when *a* and *b* are similar, identical items *a* lead different groups of participants—regardless of whether they performed a categorization or a decision-making task—to evaluate slippery slope arguments as strong and to categorize new items, *b*, as *F*, when *a* had been categorized as *F*. However, this did not happen when *a* and *b* were dissimilar. When *a* had been categorized as *F* and *a* and *b* were dissimilar, the same participants, who initially rejected categorizing *b* as *F*, re-appraised this decision on being told about an intermediate item *c* that was similar to *b*, and that was also categorized as *F*.

These results show that when both the beginning and end of the argumentative chain of a slippery slope argument are similar, the probability that both were perceived as belonging to the same category is higher and hence the persuasive strength of the argument is stronger. These results suggest that the persuasiveness of the slippery slope argument is due to the concatenation of antecedents/evidence and consequences/reasons that are perceived as similar.

In conclusion, the above study shows how the concept of similarity and probabilistic tools of cognitive psychology can be used for shedding light on an old philosophical problem in argumentation, i.e., the problem of the persuasiveness of the slippery slope argument. This line of research suggests that an evidence-based, descriptive approach can be useful to move forward the traditionally more normatively oriented discussions of the Argumentation field.

## CASE 2: THE AD HOMINEM ARGUMENT

A second classical argumentative fallacy that has initiated some empirical investigation is the *ad hominem* argument. In an “*Ad hominem*” argument, it is the person who makes a statement rather than the veridicality of the statement that is attacked by the opponent. In other words, the proponent of a statement is targeted instead of the statement itself (Walton, 1998). According to van Eemeren et al. (2012), there are three variants of this fallacy: “(a) an abusive variant of *ad hominem*, in which the other party’s person is attacked directly by depicting them as stupid, bad, or unreliable, (b) a circumstantial variant, in which the other party is attacked indirectly by casting suspicion on their motives, and (c) a *tu quoque* variant, in which the other party is attacked by pointing out a contradiction in their words or between their words and their deeds” (p. 347). Recent experimental research (van Eemeren et al., 2009) has shown that participants’ judgments of how reasonable an *ad hominem* fallacy is are a function of the strength of the argument that targets the proponent. Thus, the abusive variant of the *ad hominem* argument is judged as the most unreasonable and the *tu quoque* as less so.

The fact that experimental subjects judge the abusive *ad hominem* as an unreasonable discussion move raises the question of why is it that this fallacy occurs as often in argumentative discourse (i.e., oral and written) without it being recognized as a fallacy by the audience. In other words, the unreasonableness of this fallacy is easily recognized in experiments but in real life situations this fallacy remains undetected more often than not. Recently, this question has been tested from a pragma-dialectical perspective using the concept of “strategic maneuvering” (van Eemeren et al., 2012).

### PRAGMA-DIALECTICS AND “STRATEGIC MANEUVERING”

Recent work in argumentation theory has started to empirically test the psychological concerns about the extent to which people are prone to employing procedural norms in rational argument rather than focusing solely on normative issues as traditional argumentation research does (van Eemeren et al., 2009). These studies have been conducted under the so-called pragma-dialectical account of argumentation (van Eemeren and Grootendorst, 2004). While strictly logical approaches are focused on the study of arguments as ready-made products, pragma-dialectics is developed to study the different kinds of procedural rules that define reasonable argumentation. Following this approach, the *ad hominem* argument is viewed as fallacious specifically because it violates fundamental procedural norms of rational arguments and not solely because it violates a particular norm or logical rule (as in normative theories).

Recently, pragma-dialectics has incorporated elements from rhetoric into experimental analysis of *ad hominem* argument (van Eemeren et al., 2012). In particular, the authors have raised questions regarding the nature of “strategic maneuvering” from a pragma-dialectical perspective. “Strategic maneuvering” uses “the opportunities available in the dialectical situation for steering the discourse rhetorically in the direction that serves their own interest best” (p. 151). Thus, strategic maneuvering enables the parties to maintain the persuasiveness in the discussion without neglecting the standards of the argumentation. This approach has been studied recently in the cognitive field of argumentative structures such as the *ad hominem* argument (van Eemeren et al., 2012) and the straw men fallacy (Lewiński and Oswald, 2013).

### TESTING THE ABUSIVE AD HOMINEM ARGUMENT USING STRATEGIC MANEUVERING

van Eemeren et al. (2012) studied the factors contributing for an abusive *ad hominem* attack to look less unreasonable. The authors describe the abusive *ad hominem* attacks as a mode of strategic maneuvering which takes on a reasonable appearance in real-life situations by mimicking legitimate critical reactions to authority argumentation. Thus, they hypothesized that the abusive *ad hominem* attack would be judged as less unreasonable when it is presented as a critical questioning of the authority exerted by the party under attack. In other words, abusive *ad hominem* argument (i.e., clearly fallacious) may be disguised as instances of non-fallacious versions of this argument form.

This hypothesis was tested in two experiments where participants saw a group of situations that included a contextual description followed by a dialog between two speakers. The instruction was to judge how reasonable or unreasonable they found the discussion contribution of the second speaker in the dialog by means of a 7-point scale. Importantly, in the contextual description of the dialogs, the first speaker was presented as knowledgeable about the topic under discussion.

In the first group of dialogs, an abusive *ad hominem* argument in disguise was included, where, the first speaker never argues by exerting authority. Since the arguer does not present themselves from a position of authority, these situations are referred to as disguised *ad hominem* argumentation. The next is an example of such an abusive *ad hominem* attack, presented as criticism to the authority in disguise:

The art museum is renovated and that is the reason why it has been inaccessible to the public for some time. The museum curator discusses this with a journalist.

Curator: I think the museum can be open again for the public. The building is in excellent shape now and it is perfectly safe.

Journalist: As a curator you may know about art but you are not knowledgeable about the safety of the building (p. 359).

Importantly, a group of dialogs containing a reasonable personal attack were included in the experiment. In those, a standpoint is defended by means of authority argumentation in which the speaker refers to themselves as an expert. Then, the second speaker replies by making a critical reaction to the relevant authority argumentation. The following is an example of a reasonable personal attack as a justified reaction to authority argumentation:

A divorce lawyer is talking with a friend about a criminal who is under trial

Divorce lawyer: I really think that this man will be charged with at least 12 years. As a lawyer I know these things.

Friend: You are a divorce lawyer not a criminal lawyer. Why should I believe you? (p. 359)

As predicted, the authors found that abusive *ad hominem* arguments were scored as less unreasonable in disguised dialogs as compared to situations where the first speaker refers to themselves as an expert. In fact, while the abusive attacks were judged as an unreasonable argumentative move when the arguer had exerted authority, their counterparts in situations where the authority was disguised were considered neither reasonable, nor unreasonable.

In conclusion, when the *ad hominem* argument is presented as a criticism to straightforward arguments of authority, it is perceived to be less reasonable. This study shows that pragma-dialectical account is starting to take into account more contextual, ecological and daily-life settings for studying argumentation experimentally. This approach stands in contraposition to the classical Argumentation research, which focuses solely in normative issues.

## DISCUSSION

### PSYCHOLOGY OF ARGUMENTATION AS AN INTEGRATIVE SCIENTIFIC ACCOUNT

In this article, we have focused on the Bayesian analysis of the slippery slope argument and the pragma-dialectical analysis of the *ad hominem* argument in order to exemplify the merits of the experimental approach for describing the cognitive mechanisms of argumentation.

However, a general point to clarify is whether psychology of argumentation is either (a) a new perspective on argumentation, combining both normative and descriptive elements, or (b) a descriptive approach in opposition to the normative stances of logic, rhetoric, and dialectic. We claim that psychology of argumentation is an integrative scientific account. It is neither a new perspective nor a combination of perspectives. In fact, psychology of argumentation possess descriptive elements and also recognizes the necessity of normative accounts when, for instance, epistemic vigilance (see Sperber et al., 2010; Mazzarella, 2013; Padilla Cruz, 2013) is required as a consequence of the effectiveness of certain fallacies.

The quest for a more complete explanation of the concept of fallacy in order “*to bring the normative dimension better into relation with the psychological dimension*” (Walton, 2010, p. 160) is not new. For instance, Walton (2010) explores the possibility of elucidating the misleading nature of many informal fallacies of reasoning in terms of their connections to cognitive heuristics (Walton, 2010; but see also Correia, 2011). Walton’s approach postulates argumentative heuristics without using recent cognitive psychology research to support his view. A heuristic is a *mediating concept between the notion of fallacy and ‘retractable argumentation'* (Walton, 2010). To explain this mediating role, Walton introduces the notion of a parascheme, *a device that can be used to represent the structure of a heuristic as a fast inference instinctively linking a conclusion, and that is commonly used to make decisions* (Walton, 2010).

In this light, producing a fallacy is not about doing something inherently “wrong,” but rather the result of not selecting the optimal strategy given the circumstances. A genuinely cognitive explanation of fallacies, therefore, must not only explain how these biases operate, but also specify the conditions under which they operate and become argumentatively and epistemically disadvantageous (Oswald and Maillat, 2011). Oswald and Maillat (2011) hence argues that the study of fallacies also needs a normative dimension, which helps identify clear criteria to distinguish consistent from fallacious arguments.

For Mercier and Sperber (2011) the role of argumentation is not truth seeking, but rather helping defend a point of view. In other words, argumentation plays essentially a psychological function. Still, quite a few argumentation theorists sustain the opposite view. For example, Morado (2014) states that “*if a bad argument is convincing* [it]* is precisely because it appears to help find the truth.”*

Mercier and Sperber (2011) consider the evolution of reasoning is linked to the evolution of human communication. Reasoning allows humans to produce arguments to convince recipients in accepting or trusting what they are told. And at the same time, it allows recipients to assess the strength of these arguments and accept valuable information that would otherwise be suspicious (Mercier and Sperber, 2011; as a cautionary side note, see Navarrete and Santamaría, 2011 for a comment on why such evolutionary arguments should be treated with special care). Despite the obvious relevance of cognitive perceptions to the study of argumentation, research on cognitive aspects of reasoning (and by extension those of argumentation) has traditionally been kept within the limits of cognitive psychology, from Wason seminal works in the 1960s (Wason, 1960, 1966) and the pioneering work of Tversky and Kahneman (1974) on cognitive heuristics.

In this sense, Mercier and Sperber (2011) proposal is close to that of the rhetorical perspective to argumentation. It understands argumentation as a natural process of persuasive communication (Wenzel, 1990). Sperber et al. (2010) argue that humans “*have a set of cognitive mechanisms for epistemic vigilance, at risk of being misinformed by others*” (p. 359). These cognitive filters are taken to monitor incoming information and calibrate confidence in their source while simultaneously evaluating the consistency of the message. Such a role is akin to the fallacies associated with the source in a theoretical framework in which the rhetorical effectiveness is seen as a product of cognitive limitations and biases (Hart, 2011; Oswald and Maillat, 2011).

To summarize, the psychology of argumentation could be defined as a research program involving a dual-process account of reasoning and Bayesian reasoning representation systems as models that provide an explanatory framework for interpreting the rhetorical effectiveness of fallacies. Fallacies can be characterized by the kind of consequences that lead to epistemic vigilance (Sperber et al., 2010). Hence, we can differentiate the psychology of argumentation as a separate field as opposed to a particular cognitive approach, or a philosophical logic-based and apriorist stance against the preponderance of the evolutionary grounded search for truth.

### A THEORETICAL FRAMEWORK FOR THE DESCRIPTIVE AND NORMATIVE ACCOUNTS OF ARGUMENTATION

In Argumentation, the mechanisms underlying persuasive arguments have been traditionally studied by employing philosophical accounts (e.g., rhetoric, dialectics, and logics). Furthermore, these philosophical accounts have traditionally postulated models of argumentation based on an *idealization* of the phenomena (Hansson, 2007). Thus, we can distinguish two types of idealization: (1) a “simplified idealization” which neglects several relevant aspects of real life complexity; or (2) a “perfectionist idealization” which attempts to satisfy higher rationality standards than those that are actually affordable by real agents.

Following this idea, the type of idealization of the normative approaches would fit in the first category, i.e., a simplified, reductionist idealization. Since their theoretical distinctions are made on a constrained, normed language (e.g., the “fallacious” character of an argument is because it violates a logical rule), normative views neglect the cognitive complexities of the agents involved in a real, spontaneous argumentative discussion. Here, we have shown how cognitive models and probabilistic tools are starting to take into account these complexities by embracing a more analytical and descriptive account of argumentation.

The experimental account we advocate here is in line with the so-called “practical approach of logical reasoning” of Gabbay and Woods (2003). The idea of a practical logic of reasoning is based on the description of a set of behavioral aspects of practical agents under particular cognitive conditions. In Gabbay and Woods (2003) words:

A cognitive agent is a being capable of perception, memory, belief, desire, reflection, deliberation, decision and inference. A practical cognitive system is a cognitive system whose cognitive agent is a practical agent in our sense, that is, an individual. A practical logic gives ‘a certain kind of description’ of a practical cognitive system. (p. 7)

In this view, a cognitive system can be defined as a 3-tuple: a cognitive agent, cognitive resources, and cognitive tasks performed dynamically in real time. This 3-tuple represents a plausible cognitive model for describing argumentative structures such as the slippery slope argument and the *ad hominem* argument. First, a cognitive agent or agents are present (the speakers). Second, these agents perform a task in real time by evaluating the persuasiveness of the slippery slope argument or the degree of unreasonableness of the *ad hominem* argument (e.g., they are being influenced by perception, memory, beliefs, desires, deliberation, decision, and inference). Third, other cognitive tasks are involved in the evaluation process, such as comparing the similarity between antecedents in an argumentative structure in the case of the slippery slope (Corner et al., 2011); or judging a personal attack as less unreasonable when the *ad hominem* argument is presented as criticism against arguments by hidden authority (van Eemeren et al., 2012).

The above model allows for what Woods (2013) called the “naturalization of logics.” The two main components of this research program are *heavy-equipment mathematics*, i.e., more powerful mathematical techniques available for representing knowledge (e.g., the formalisms of normative theories); and cognitive models promoting a naturalist description of the argumentative phenomena (e.g., the Bayesian and pragma-dialectics experimental accounts).

In terms of our proposal, this approach is particularly useful since it represents a potential common framework in which cognitive and normative accounts in psychology can converge.

## CONCLUSION

Here we show how descriptive approaches can shed light on the psychological mechanisms of argumentation by analyzing experimental evidence related to two classical argumentative structures. Furthermore, we argue that psychology of argumentation provides an integrative scientific perspective unlike normative or aprioristic approaches. This integrative approach brings a wide swath of aspects of psychological literature (e.g., emotions, decision making) into a single comprehensive framework, re-conceptualizing classical rationality in a framework that allows for experimental testing (e.g., using Bayesian theory). All in all, we believe employing more descriptive and experimental accounts of argumentation would help Psychology to “keep on” bringing the cognitive and normative accounts of argumentation closer, with the final goal of establishing an integrated area of research on the psychology of argumentation.

### Conflict of Interest Statement

The authors declare that the research was conducted in the absence of any commercial or financial relationships that could be construed as a potential conflict of interest.
